# Editorial: Feminine, feministic, feminists, and feminisms

**DOI:** 10.3389/fsoc.2025.1645571

**Published:** 2025-07-02

**Authors:** Sadia Irshad, Bushra Naz, Abida Sharif

**Affiliations:** 1 Air University Islamabad, Islamabad, Pakistan; 2 Prince Sattam Bin Abdulaziz University, Al Kharj, Saudi Arabia; 3 Fatima Jinnah Women University, Rawalpindi, Pakistan

**Keywords:** feminine, feminisms, feministic, feminists, gender

## Introduction

Globally, an estimated 736 million women—nearly one in three—have experienced physical or sexual violence in their lifetimes (World Health Organization, 2024), and economic participation of women sees slow progress; significant pay and opportunity gaps remain globally, especially in labor force participation and income (World Economic Forum, 2023). Meanwhile, political representation lags at roughly 27% of parliamentary seats held by women, and anti-gender backlash is on the rise (Human Rights Watch, 2023). In this context, a pluralist, intersectional feminism gains urgency: one that attends to local struggles while recognizing global links. Global Feminism—a dynamic and multifaceted evolving constellation of struggles—seek to dismantle genderized, religionized, emotionalized, racialized, colonized, regionalized and digitized forms of asymmetrical domination. While the early histography of feminism often privileges the “waves” model rooted in Euro-American contexts— ranging from the first wave's focus on suffrage to the second's equality and reproductive rights, from third wave's identity and intersectionality to fourth's digital activism—global feminism spans across these temporal and geopolitical boundaries. From its inception, global feminism has sought to forge solidarities across borders while grappling with critiques of Eurocentric and the hegemonic tendencies of Western feminist discourse and a decolonized feminist praxis attentive to the specific socio-historical contexts of women in Global South. Contemporary global feminism is deeply intersectional, transnational and anti-capitalist in orientation. It urges us to reconcile the politics of recognition, redistribution and revision of women's rights. It continues to prioritize the urgency of addressing systemic violence, climatic stewardship, bodily autonomy, mobility and digital representation.

In exploring the continuum from the feminine to feminism, this Research Topic—aptly titled *Feminine, feministic, feminists, and feminisms*—reveals how diverse approaches converge on the pursuit of gender justice. The purpose of this Topic is to offer authors and readers an enriching opportunity to deepen their knowledge of global feminism along with the cultural, ethnic, digital, legal, personal and philosophical dimensions of women engaging with intersecting fields of aesthetic, ethics, morality, positionality, intellectual traditions, ideology, oppression, patriarchy, and sexuality.

This Research Topic of 13 essays aims at bringing together research writings across various disciplines to deliberate on how women as philosophical, aesthetic, political and religious categories are represented, negotiated, and transformed in today's lifeworld. The essays are broadly categorized under five interrelated themes highlighting local struggles and global solidarities.

## Gendered-labor and the political economy of mental health: contradictions in neoliberal subjectivities

Following three essays “*Work-Family Conflict, Overwork and Mental Health of Female Employees in China”* (Ma et al.), “*Career Women's Mental Wellbeing in the Era of Population Decline”* (Zhou et al.), and “*What's in Store for Females After Breaking the Glass Ceiling?*” (Cheng and Wang) interrogate the gendered contradictions of neoliberal capitalism, where discourses of empowerment coexist with the intensified precarities in both professional and domestic spheres. Drawing from feminists' political economy and sociology of work, the authors critically examine how overwork, shrinking population regimes and post-glass-ceiling dynamics recalibrate women's labor as sites of emotional exhaustion and silent suffering. Mental wellbeing emerges as a contested terrain, not merely individual but shaped by structural asymmetries and symbolic violence embedded in masculine corporate cultures. These essays unfold the complicated idea of meritocracy on the one hand and reveal the double burden and psychic costs of navigating gendered institutions under global capitalism at the other hand.

## Existence and resistance across borders: feminist spatiality

Essays “*Feminism in the Borderscape: Juarense Women Against Injustice”* (Mehan and Dominguez), “*Informal and Revolutionary Feminist Placemaking”* (Mehan), and “*A Psycho-ethnography of the Self at #PoWESconf”* (Silverio), foreground feminist spatial theory and sociology of place to explore how women contest and reimagine spatial orders. From the militarized borders of Ciudad Juárez to informal feminist urban interventions and academic conference spaces, the authors demonstrate how gendered space is both constructed and contested. Drawing on Doreen Massey's conceptualization of space as relational and dynamic, and the notion of borders as sites of both exclusion and potentiality, these studies highlight women's spatial agency in the face of structural and epistemic violence. The psycho-ethnographic lens further introduces a reflexive turn, mapping the emotional geographies and aging of feminist selves within institutional setting.

## The dynamics of embodied agency, reproduction and leisure: feminist health rights

Centered on the feminist health studies and Sociology of body, essays “*The Need for Assisted Reproductive Technology Regulations: A Case for Women in the Philippines”* (Biana)*, “Befriending the Body Through Clothes: The Role of Clothing in Secular and Religious Women's Body Appreciation”* (Stolovy), and “*Young Women's Leisure Time Physical Activity (LTPA) Determinants: A Mixed Methods Approach”* (Fernandez-Lasa et al.) reveal socio-religious and bio-technological regulation of women bodies—whether through state of religion and secularism, aesthetic regimes or public health discourses. These essays interrogate how clothing, movement, leisure and technological advancements become entangled with individual identity, religious agency, and state control. The call for ART regulation, for instance, speaks to broader questions of reproduction justice, while the other studies foreground bodily autonomy as personal act situated within normative regimes of femininity. Far from being passive recipients, women emerge as negotiators of embodied subjectivity, enacting their agency within and against the dominant frameworks.

## Politic, prejudice and masculinities: contested feminism

Essays “*Attitudes of Women Politicians Toward Their Roles in Politics: Northern Cyprus”* (Artaç and Ogurlu), and “*A New Variation of Modern Prejudice: South Korean Young Men's Anti-Feminism and Male-Victim Ideology”* (Jung) examine the emerging counter-narrative of anti-feminism masculinities within the domain of feminist political ideology and sociology of emotions. How do women in formal politics navigate gendered expectations, performative roles, and institutional inertia. Essay in south Korean context unpacks a growing ideological backlash among young men characterized by male victimhood discourse and ressentiment. Recognition of systematic gender hierarchies offer a nuanced critique of political subjectivity in a time marked by populism, re-traditionalization, and the contested legitimacy of feminist gains.

## Intimate violence, memory and portrayal: the intersectional feminism

Essays on “*Differences in Help-Seeking in Intimate Partner Violence: Jewish and Arab Women in Israel”* (Ne'eman-Haviv and Shafran) and “*Shoah Pornography: The Stalag Phenomenon in Israel in the 1960s,”* delve into the sociology of violence, ethnic stratification and memories through an intersectional lens. The essays addresses the questions, i.e., how do social capital, cultural scripts, and state relations mediate access to health care and protection? How differential help-seeking behavior may vary across ethnonational lines? Evidence from both research essays engage with historical trauma and representational violence. Both essays confront the haunting persistence of gendered trauma in public and private spheres.

Together, these essays reflect the conceptual breadth and methodological richness of contemporary feminist sociology. Research Methodologies particularly from Global South rely heavily on the extended case method. They speak across disciplines, geographies, and positionalities, yet converge in their shared commitment to interrogating power, disrupting normative knowledge systems, and imagining emancipatory future of feminism.

## Conclusion: from feminine roots to feminisms reimagined

*Feminine, feministic, feminists, and feminisms* offers a pluri-vocal atlas of gendered power and resistance. By weaving together studies on work, body, violence, and identity, it demonstrates how feminisms evolve—from the feminine as everyday practice to feminist movements that traverse borders. The title encapsulates this arc: acknowledging feminine experiences, interrogating feministic frameworks, amplifying feminist actors, and envisioning new feminisms. Together, these contributions chart pathways toward justice, solidarity, and transformative change for all women. A crucial part of this Research Topic is to underscore that feminism is not owned by any one identity. Men can be feminists. Non-binary individuals can be feminists. Feminism is not a closed ideology but an evolving conversation rooted in the desire for equity and dignity. The editorial team was especially keen to include contributions that demonstrate how feminism must adapt to new forms of injustice—from algorithmic bias to ecological precarity—while remaining anchored in a commitment to care, equity, and justice.

We invite readers, scholars, and activists alike to engage deeply with the following contributions and to reflect on three questions:—Where do gender inequalities still persist in your discipline, institution, or context?—What feminist responses have proved most effective—or most limited?—How might we imagine a more just, inclusive, and feminist future?

This Research Topic is a testament to the fact that the political is still personal, and the personal remains profoundly political. It insists that feminism is neither obsolete nor fully achieved—but ongoing, multivocal, and transformative. By tracing experiences from everyday life to the institutional spaces, notable strides toward gender equality and advancement of women in leadership roles, much remains to be done to fully acknowledge and valorize the diversity, complexity, and contextual specificity of women's lived experiences. In doing so, this Research Topic contributes to a global and pluralist feminist praxis—attuned to difference, embedded in context, and radically committed to justice and inclusivity leaving no one behind.
