# Correction: Editorial: Feminine, feministic, feminists, and feminisms

**DOI:** 10.3389/fsoc.2026.1804852

**Published:** 2026-03-10

**Authors:** Sadia Irshad, Bushra Naz, Abida Sharif

**Affiliations:** 1 Air University Islamabad, Islamabad, Pakistan; 2 Prince Sattam Bin Abdulaziz University, Al Kharj, Saudi Arabia; 3 Fatima Jinnah Women University, Rawalpindi, Pakistan

**Keywords:** feminine, feminisms, feministic, feminists, gender

The funder “Prince Sattam bin Abdulaziz University project number (PSAU/2025/R/1447)” was erroneously omitted.

The correct **Funding** statement is given below.

**Funding**

The author(s) declared that financial support was received for this work and/or its publication. This study was supported by Prince Sattam bin Abdulaziz University project number (PSAU/2025/R/1447).

The original version of this article has been updated.

